# On the Right Track? Investigating the Effect of Path Characteristics on Visuospatial Bootstrapping in Verbal Serial Recall

**DOI:** 10.5334/joc.2

**Published:** 2017-12-08

**Authors:** Anthea Allan, Candice C. Morey, Stephen Darling, Richard J. Allen, Jelena Havelka

**Affiliations:** 1 Department of Psychology, University of Edinburgh, GB; 2 Memory Research Group, Queen Margaret University, Edinburgh, GB; 3 School of Psychology, University of Leeds, GB

**Keywords:** working memory, spatial memory, serial recall, visual short-term memory, path complexity

## Abstract

Visuospatial bootstrapping (VSB) occurs when memory for verbal material is enhanced via association with meaningful visuospatial information. Sequences of digits are visually presented either in the center of the screen or within a keypad layout in which the digits may be arranged identically to familiar pin pad and mobile phone layouts, or randomly. Recall is consistently higher when digits are presented in the familiar layout. This “bootstrapping” could involve primarily long-term knowledge of the layout, primarily short-term memory of the unique spatial path, or may depend on both. We manipulated the path complexity of sequences to test whether the VSB effect depends on the quality of spatial representations in conjunction with the familiarity of the spatial layout in two experiments. We consistently observed both VSB effects and path complexity effects on verbal serial recall, but never observed any interaction between these factors, even when articulatory suppression was imposed. Analysis of recall by serial position revealed that the VSB effect was focused on the end-of-list items. Our finding of pervasive path complexity effects on verbal serial recall suggests incidental encoding of spatial path occurs during visually-presented verbal tasks regardless of layout familiarity, confirming that spatial factors can affect verbal recall, but ruling out the notion that incidental spatial paths are uniquely and voluntarily encoded with familiar layouts.

The term “bootstrapping” conventionally refers to the improvement of one’s situation by personal effort. “Visuospatial bootstrapping” (VSB; Darling, Allen, & Havelka, 2017), a phenomenon identified by Darling and Havelka (2010), applies this notion to maintenance in working memory, which is known to be severely limited (Cowan, 2001). In this context, bootstrapping describes an advantage accruing to the recall of verbal items through their association with meaningful visuospatial information stored in long-term memory. There is abundant evidence that verbal working memory can aid execution of visuospatial working memory tasks (Brown, Forbes, & McConnell, 2006; Brown & Wesley, 2013; Pearson, Logie & Gilhooly, 1999). Darling and Havelka (2010) investigated the possibility of the obverse contingency. They displayed sequences of to-be-remembered digits on-screen in different formats, each arrangement affording a different degree of potentially useful spatial information. These included 5–8 digits presented singly in the center of the screen, within a linear display, or in a matrix replicating the format of the familiar, typical keypad of telephones or automated teller machines. Spoken recall of the verbal list was significantly higher in the typical keypad condition than in either of the other conditions. This familiar keypad benefit has been replicated several times, confirming that presenting verbal materials within a familiar spatial layout consistently improves recall (Allen, Havelka, Falcon, Evans, & Darling, 2015; Calia, Darling, Allen, & Havelka, 2015; Darling, Allen, Havelka, Campbell, & Rattray, 2012; Darling, Parker, Goodall, Havelka, & Allen, 2014). However, it is uncertain whether VSB effects depend mainly on commandeering long-term memories or alternatively, on commandeering domain-specific visuospatial processes for the benefit of verbal serial recall. Our experiments are intended to address this gap.

Currently, there is evidence favoring both potential sources of the VSB effect, but for a VSB effect to occur in verbal recall, the spatial layout in which the digits are presented must be known to the participants. Darling et al. (2012) demonstrated this by testing for VSB effects with stimuli that preserved the spatial layout of the standard keypad while removing the long-term digit-position associations. In addition to the typical keypad and central presentation conditions, Darling et al. (2012) included conditions with novel keypad displays. Sometimes the same novel configuration was shown throughout a block, whereas in other blocks, the digit configuration changed randomly in each trial. The VSB effect was observed for the typical keypad display, but not for either the static or the changing novel keypad display. Since the typical keypad and the novel keypads differed only in familiarity, the authors suggested that long-term memory representations were the primary factor producing the VSB effect. An exploratory finding that performance in the novel static keypad, originally the worst of all conditions, improved over the session provided further confirmation. Darling et al. (2012) suggested the early inferior performance could be attributable to overcoming initial interference due to the long-term knowledge of the typical keypad. Alternatively, supposing that the participants became aware of a different regular display, it could be that an initial attentional cost was incurred in the endeavor to learn the new layout, but once learned, a VSB effect eventually emerged. In any case, this pattern of results suggested that the VSB effect does not merely depend on presenting the digits in distinct locations, but depends on prior knowledge of the bindings between locations and digits.

Further evidence that long-term knowledge of the layout is essential for producing bootstrapping comes from developmental research (Darling, Parker, Goodall, Havelka, and Allen, 2014). Darling et al. (2014) tested verbal recall of visually-presented digits in one group of 6-year-old children and another of 9-year-olds in addition to young adults. Participants viewed to-be-remembered digits in three formats: single display, typical keypad and novel keypad. A VSB effect comparable to that in adults emerged in the 9-year-old participants, but not in the 6-year-olds. That older children, who have likely accumulated more experience using devices with the typical keypad layout, show the VSB effect while younger children do not is consistent with the notion that the VSB effect depends on accessing long-term knowledge. In contrast, Calia, Darling, Allen, and Havelka (2015) documented evidence of the resilience of the VSB effect against impairing effects of aging on memory, which is consistent with the notion that the VSB effect relies on long-term memories that are unimpaired in healthy older adult populations. However, interpretation of the finding that young children do not evince VSB effects is not straightforward, because children around 6 years old may also differ from older children in their ability to maintain visuospatial paths (Schumann-Hengsteler, Strobl, & Zoelch 2004). Schumann-Hengsteler and colleagues found that visuospatial order recall in 10-year-old children was sensitive to path complexity manipulations, which are believed to arise due to domain-specific representation of the visuospatial image. Spatial order recall in 6-year-old children was not impaired with complex paths, leaving open the possibility that 6-year-olds differ from older children and adults not only in the strength of their long-term visuospatial knowledge, but also in that they do not spontaneously apply rehearsal for the encoding and maintenance of spatiotemporal information (see also Morey, Mareva, Lelonkiewicz, & Chevalier, in press). If this is indeed the case, it could as easily explain the absence of VSB effects in young children without assuming that the youngest children lacked long-term knowledge of the familiar keypad layout.

Even if the VSB effect is not driven merely by the presentation of verbal information in distinct locations, there is other evidence suggesting that domain-specific spatial processes do contribute to the VSB effect. Allen et al. (2015) employed classic dual-task designs to investigate whether verbal and visuospatial processes contributed to the VSB effect. Though they observed disruptive effects of articulatory suppression on digit recall, this disruption was not greater with typical keypad than with central presentation. In fact, disruption was attenuated in the familiar keypad condition. This suggests that meaningful spatial information enhanced recall, perhaps exerting a protective effect counteracting any impairment from articulatory suppression carried out during encoding. Contrarily, concurrent spatial tapping selectively impaired recall in the familiar keypad condition, but only when spatial tapping was carried out during encoding of the digits, not during recall. If one assumes that spatial tapping selectively disrupts the use of some domain-specific visuospatial short-term memory process and articulatory suppression selectively disrupts use of a comparable verbal process, then this series of findings suggests that domain-specific visuospatial processes must be available during encoding for the VSB effect to emerge. Allen et al. argue that this evidence suggests that information available in domain-specific verbal and spatial processing streams comes together during encoding, producing the VSB effect.

Thus far, the available evidence provides two necessary conditions for producing the VSB effect: presentation of information in unique spatial locations, and prior knowledge of the binding between verbal information and spatial layout. However, some evidence is puzzlingly inconsistent with these stipulations. Darling and Havelka (2010) did not observe a VSB effect for recall of digit sequences presented along a linear number line. This presentation presumably afforded both criteria that seem necessary for producing VSB effects, yet no effect emerged. Darling et al. (2012) subsequently suggested that the absence of the effect could be due to potential interference resulting from following a spatial path through the 1 × 10 linear display, and the consequently overlapping path crossings which would have resulted (Darling, Allen, Havelka, Campbell & Rattray, 2012). If this assessment is correct and the quality of the visuo-spatial representation is a factor in the VSB effect, then the VSB effect should be attenuated when digit sequences with complex spatial paths through the familiar keypad must be recalled. However, this supposition has never been formally tested, nor has path complexity ever been assessed or controlled for in a VSB paradigm.

In fact, the impact of spatial path complexity and other organizational factors on recall of spatial sequences has largely been overlooked even in the spatial serial memory literature (Hurlstone, Hitch, & Baddeley, 2014; Parmentier, Elford & Maybery, 2005). This criticism is founded in the irregularities in procedure and inconsistent results historically demonstrated in the Corsi Blocks tapping test (CBTT). In this test, the administrator taps sequences of varying lengths on a pre-constructed set of blocks, and the participant attempts to reproduce the sequence identically (Corsi, 1972). The CBTT has been criticized for non-standardized administration, different criteria for termination, differences in the source of stimuli, and indifference to the spatial relationships between serial items which determine the sequence paths (Berch, Krikorian and Huha, 1998). Subsequent research investigating the effects of variations in the configuration of spatial sequences, such as vertical or horizontal symmetry (Rossi-Arnaud, Pieroni, & Baddeley, 2006), shows that manipulating organizational factors produces consistent effects on recall success that should be taken into consideration.

Among the clearest of these regularities are path complexity effects, where recall accuracy is a function of the number of times the ideal line joining the spatial positions crosses itself. “Complex” paths with many crossings are recalled less accurately than “simple” paths with fewer or no crossings (Orsini, Pasquadibisceglie, & Picone, 2001; Busch, Farrell, Lisdahl-Medina & Krikorian, 2005; Smirni, Villardita & Zappala, 1983). For example, Parmentier et al. (2005) manipulated path complexity in terms of path crossings and their angles. Path presentation occurred in the form of dots appearing briefly, singly, and sequentially on a screen. Within each sequence, zero, three, or six crossings could occur. Dots were then presented together in their correct locations for the participant to reconstruct the correct order in which they had been presented. This de-emphasized memory for item information, maximized demands on order memory, and avoided the rigidity of typical CBTT (Parmentier et al., 2005). Complexity of path configuration indeed reduced recall success. Path crossings appeared to do their damage during encoding of the sequence, because the negative impact of multiple path crossings on recall was unaffected by imposing a retention interval (Parmentier & Andrés, 2006). Moreover, Kemps (1999, 2001) considered how complexity of a to-be-remembered path limits visuospatial short-term retention, suggesting that the configuration of some paths can optimize recall performance. Using a form of the CBTT, Kemps manipulated structural complexity, defined in terms of the Gestalt principles of symmetry, repetition, and continuation: paths which incorporated one or more of these features were regarded as “structured”. The elements of a structured path were interdependent and predictable, thereby reducing complexity and exhibiting redundancy (Kemps, 2001). A structured path follows a regular order, therefore long-term memory representations could be facilitating short-term storage of visuospatial information. Serial recall was found to be better for structured as opposed to unstructured sequences, and this difference was resistant to a secondary task of spatial tapping.

It is not clear though that path complexity would impact recall of verbal information at all, even if the verbal information were presented in distinct spatial locations through which paths could be drawn. Guérard, Morey, Lagacé, and Tremblay (2013) manipulated path complexity to investigate the asymmetry of binding between verbal and spatial features in serial memory. To-be-remembered sequences comprised seven either phonologically similar or dissimilar letters, displayed in various locations around a screen. Participants could be required to recall either locations or letter identities in order. Though phonological similarity impaired both verbal and spatial task performance, spatial complexity impaired memory for location order but not letter order. These findings suggest that verbal serial order memory is impervious to incidental path complexity effects. However, this evidence does not necessarily mean that path complexity would have no impact on recall of verbal serial order for stimuli with such over-learned mappings as the digit-location pairings used in the VSB paradigm. Indeed, if encoding and maintenance of a visuospatial sequence is a necessary condition for producing the VSB effect, then we expect that path complexity *must* impact verbal recall in this case. If redundant encoding of the visuospatial sequence is causing the verbal recall enhancement observed in VSB, then providing a complex sequence that is more difficult to remember correctly should reduce the size of the VSB effect, or perhaps even annihilate it, just as Allen et al. (2015) observed by imposing spatial tapping during bootstrapping.

We report two experiments investigating the resilience of visuospatial bootstrapping to the effect of path complexity. Consistently with Guérard et al. (2013) and Parmentier et al. (2005), we used path crossings to vary path complexity, creating unique lists to be used when presenting stimuli in typical and random keypad layouts that contained zero or several path crossings. If the VSB effect is due to the encoding and maintenance of visuospatial sequences alongside the to-be-recalled verbal ones, then it should diminish with complex paths. We should observe not only that path complexity effects occur in the VSB paradigm, but that the normal keypad advantage diminishes with complex compared to simple paths. We included a random keypad display condition to provide a fair comparison for judging whether the VSB effect disappeared in the typical display condition due to path complexity, which can be manipulated equivalently in typical and random displays. In addition to the list-wise response accuracies that are normally reported in studies of the VSB effect, we report accuracy as a function of input serial position to observe at which points in the list increased path crossings reduce accuracy, and at which points the VSB effect emerges. This provides an additional indicator of whether path crossings are changing the VSB effect, or whether path complexity and display format are both affecting digit recall.

## Experiment 1

### Method

***Participants.*** Thirty-four students (6 male) attending the University of Edinburgh were recruited via the online student jobs database. All were naive to the visuospatial bootstrapping phenomenon. Participants provided written consent indicating their agreement to take part in our study, which was approved by the local ethics committee. Ages ranged from 19 to 43 (*M* = 25.71, *SD* = 5.96). All reported normal or corrected-to-normal sight, and each received an honorarium of £7 in return for participating.

***Apparatus and stimuli.*** The participants’ task was to memorize visually-presented digit sequences and recall aloud those digits in their correct presentation order. Testing occurred individually in a session lasting approximately 60 minutes. Sessions were conducted in a private booth equipped with two desks situated side-by-side. The participant sat at the right-hand desk on which a computer was placed. The researcher sat at the left-hand desk to record the participant’s responses. A tall screen between the desks allowed the participant a degree of privacy from the researcher seated nearby. The experimental program was implemented using E-Prime, (Schneider, Eschmann, & Zuccolotto, 2002; version 2.0.10.242). It was installed on a Dell Optimex 790 computer system with 8 GB RAM, a 17-inch screen set to a 1024 by 768 pixel display. The middle cell of an image of a keypad matrix was centered on the screen. The individual cells of the matrix, measuring 60 by 60 pixels each, were each separated from the outlined boundaries of neighboring cells by 12 pixels. All digits were presented in black. As a participant’s familiarity with the layout of a mobile telephone keypad was a significant factor in the experiment, the computer’s keyboard was placed out of sight for the duration of the session. The researcher controlled the progression of the program using the mouse.

In any single block, eight sequences of seven digits were presented in each of three display types. In the normal keypad display, a 3 × 3 + 1 matrix replicated the detail of the familiar keypad used on mobile phones and automated teller machines. Alternatively, in the random keypad display the digits 0 to 9 were shown in novel positions in a similar 3 × 3 + 1 arrangement. This arrangement (see Appendices) was used throughout the sessions. In the central display, single digits were displayed in the central cell of an otherwise unfilled keypad matrix.

Within the eight normal or random display trials per block, four sequences were designed to trace a simple path and four to trace a complex path. Complexity of path configuration was defined according to the number of times the imaginary connecting path taken by the sequential progression of numbers around the keypad intersected itself (Kemps, 2001; Parmentier et al, 2005). Complex path configurations were sequences of digits characterized by either 3, 4, or 5 intersections. The average number of intersections per sequence in the normal and random keypad conditions were equivalent (*M*s = 3.75). Simple sequences had no intersections. To generate appropriate sequences, we collected randomly-generated sequences from a separate VSB study carried out the previous year by a student project group in which all sequences were randomly generated at run time. From these, sequences with 0 or 3 or more crossings assuming the normal or the random keypad were identified and selected for inclusion. The complex and simple sequences for the normal and random keypad conditions are given in Appendices A. E-Prime randomly selected digit sequences in the central condition, for which no variation in path configurations was possible.

***Procedure.*** A complete experimental session comprised presentation of 72 sequences. The session was subdivided into three blocks of 24 trials, with a compulsory break of at least one minute between each block.

With the participant seated in front of the computer, the researcher commenced the experimental process by clicking the mouse. On-screen instructions outlining the procedure were presented to the participant. Three practice trials followed, one in each presentation type. On the participant’s acknowledgment of readiness, the researcher started the trial process. Each trial began with a fixation cross appearing on the screen for 1000 ms. The screen then went blank for 250 ms. After this, a keypad with one digit highlighted in teal contrasting the grey background appeared for 750 ms followed by 250 ms period in which the keypad remained onscreen without any digit highlighted. This alternating routine took place seven times, displaying each digit of the to-be-remembered sequence individually. The order of presentation of sequences, both according to their keypad-type and to their path configuration, was randomized within blocks. On completion of each sequence presentation, a screen appeared instructing the participant to immediately recall that sequence aloud in the order in which the digits were presented. The researcher recorded the participant’s original replies by hand on a prepared chart, and discouraged participants from attempts to correct themselves. When both the researcher and participant were ready to continue, the researcher clicked the mouse to continue to the next trial.

To assess development of familiarity with the random keypad layout during the session, at the end of the session the participant attempted to complete the digit details on paper copies of blank templates of a keypad, first for the normal keypad and then on a separate sheet for the random keypad. Participants were then debriefed and asked to divulge any strategies they had used to aid their prospects of correct recall, which the researcher summarized in her log.

The participants’ hand-recorded recall responses were later entered into a spreadsheet independently by two research assistants. These were compared and discrepancies were resolved by a third rater. Responses were then merged with the E-Prime output and recall accuracy was computed.

***Analyses.*** We analyzed several dependent variables computed from participants’ responses to assess whether path complexity might influence VSB effects. First, we replicated previous reports of VSB phenomena by analyzing list-wise recall accuracy. We also report results in terms of number of correctly recalled items per list and as a function of serial position. We adopted this approach because we did not observe the interaction between display condition and path complexity that would confirm that path complexity only impairs verbal serial recall in the familiar layout condition, and wanted to ensure that this interaction did not emerge with alternative scorings.

We analyzed these data with Bayes factor ANOVA (Rouder, Morey, Speckman, & Province, 2012), implemented with the R package *BayesFactor* (version 0.9.12-2; Morey & Rouder, 2015) using the default prior settings. Bayes factors afford a more straightforward interpretation of the likelihood of the presence or absence of effects or interactions given the data than traditional analyses. For each analysis, we specified the potential factors in the ANOVA model, estimated models containing every possible combination of those factors, and reported which factors were present in the best-fitting model. We then assessed each factor by comparing the Bayes factor of the best model with the Bayes factor associated with the model adding or omitting the term of interest. This comparison yields a Bayes factor that summarizes the extent to which an observer’s opinion about that model term should change based on the data. Bayes factors of 1 indicate equivalence of the two hypotheses being compared. Bayes factors larger than 1 represent evidence for the alternative hypothesis and Bayes factors less than 1 represent evidence for the null hypothesis. Besides the benefit of enabling consideration of null outcomes (which is impossible with traditional methods), an important advantage of Bayes factor analyses over *p*-values is that Bayes factors are intended to be interpreted continuously. Bayes factors further from 1 afford progressively stronger conclusions about the presence or absence of an effect. Throughout our report, we invert null Bayes factors (those with values between 0 and 1) so that we consistently describe both the presence and absence of effects as values greater than 1. Each reported analysis was run with 100,000 sampling iterations.

### Results

***List-wise recall accuracy.*** List-wise recall accuracies are shown in Table 1. We entered these values into a Bayesian ANOVA with display condition (central, normal keypad, or random keypad) and path complexity (simple and complex) as fixed factors and participant ID as a random factor. The best model (*BF* = 622.88, ±0.39%) included main effects of both factors but no interaction. Inclusion of effects of display condition (*BF* > 48) and path complexity (*BF* > 26) were strongly favored. Omission of their interaction was favored by a factor of about 4. The values in Table 1 do not hint at any interaction between display condition and path complexity. Recall in the normal keypad condition (*M* = 0.65, *SD* = 0.24) was better than in the random keypad (*M* = 0.57, *SD* = 0.27) or central display conditions (*M* = 0.58, *SD* = 0.24), and recall of sequences with simple paths (*M* = 0.65, *SD* = 0.25) exceeded that of sequences with complex paths (*M* = 0.57, *SD* = 0.26).

**Table 1:** List-wise recall accuracy, Experiment 1.

Central Presentation	0.58 (0.24)	

	**Simple Path**	**Complex Path**

Normal Keypad	0.69 (0.23)	0.62 (0.24)
Random Keypad	0.61 (0.26)	0.53 (0.28)

*Note: N* = 34. Standard deviations in parentheses.

***Mean items per list correct.*** List-wise accuracy was quite variable across participants. We decided to repeat the analysis described above on the number of correctly-reported items per list, a more lenient measure. The best model (*BF* = 14,770, ±0.37%) included only an effect of path complexity. Inclusion of the path complexity effect was decisive (*BF* > 600). Omission of display condition was only marginally favored (*BF* ~ 2), but omission of the interaction was strongly favored (*BF* > 39). Participants recalled more items correctly with simple paths (*M* = 6.25, *SD* = 0.62) than complex paths (*M* = 5.98, *SD* = 0.74). Condition means are provided in Table 2.

**Table 2:** Mean number of correctly-recalled items per list, Experiment 1.

Central Presentation	5.92 (0.74)	

	**Simple Path**	**Complex Path**

Normal Keypad	6.32 (0.59)	6.05 (0.75)
Random Keypad	6.18 (0.65)	5.90 (0.73)

*Note: N* = 34. Standard deviations in parentheses. In our modelling, we used an unbalanced design in which.

**Figure 1:**
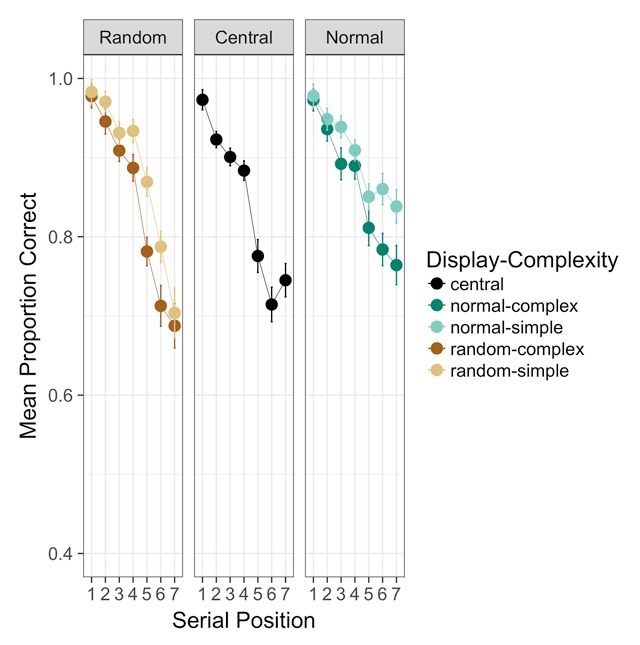
Experiment 1 serial position curves. Error bars are standard errors of the mean with the within-participants Cousineau-Morey correction applied. *N* = 34.

***Serial order analysis.*** Although the VSB effect occurs in serial verbal memory, no one has previously examined whether it occurs throughout the serial position curve. This additional analysis also provides further opportunity to compare potential differences between complexity and bootstrapping effects, which may affect recall differently depending on within-list position. Serial position curves for all conditions are shown in Figure 1. A Bayesian ANOVA on arcsine-square-root-transformed recall accuracies with serial position (1–7) as a fixed factor in addition to the factors described in the analyses reported above supported a model including effects of serial position, display condition, and path complexity, plus an interaction between serial position and display condition (*BF* = 1.45 × 10^121^, ±0.83%). Inclusion of the interaction between serial position and display condition was favored by a factor of more than 90, and inclusion of each main effect was favored by at least as much. Exclusion of the interaction between display condition and path complexity was inconclusive (*BF* = 1.17).

Though it appears obvious from inspection of Figure 1 that the typical keypad layout boosted end-of-list recall, it is not clear whether that is the only reason for an interaction between presentation layout and serial position. It is also possible that subtle differences appeared in early-list recall too. To explore this, we coded variables to reflect a few post-hoc hypothesis tests. For simplicity, we considered positions 1–4 “early-list” and 5–7 “late-list”. Late in the list, recall in the normal keypad condition appears superior to recall in the other conditions. Early in the list, the normal and random keypad conditions look quite comparable, and possibly better than central. We created new variables that either 1) allowed all three displays to differ, 2) specified that normal and random displays both differed from central presentation, 3) specified that the normal display differed from the others, or 4) specified that the random display differed from the others. We ran Bayesian ANOVAs with each of these codings of display condition combined with the simple early vs. late serial position coding, and then compared them to see which combination best accounted for the observed patterns.

The best of these models included all three levels of display condition plus an interaction between display condition and simplified serial position. Inclusion of this interaction was strongly favored over exclusion (*BF* > 46). Condition means are provided in Table 3. If there were a simple relationship between serial position and display (e.g., normal keypad better to some degree throughout the list), then the simpler coding differentiating the normal keypad display from the other conditions would have been favored, but the more complex 3-level coding of display condition was favored by a factor of 80. This suggests that the normal keypad layout’s end-of-list advantage is not the only systematic effect of display condition. The best model also distinguishes between the random and central display conditions, which apparently reflects slight benefits to recall in early and late positions with the random keypad display compared with central presentation.

**Table 3:** Mean accuracy by display condition and list position, Experiment 1.

	Early-list	Late-list

Central Presentation	0.92 (0.08)	0.74 (0.18)

Normal Keypad	0.93 (0.09)	0.82 (0.17)
Random Keypad	0.94 (0.08)	0.76 (0.20)

*Note: N* = 34. Standard deviations in parentheses. We considered serial positions 1–4 early and positions 5–7 late.

### Discussion

Experiment 1’s results revealed new clues about the nature of the VSB effect, but provided mostly tentative ideas about any unique effects of complex paths on the VSB effect. Analysis of all-or-none list-wise response accuracy revealed the typical VSB effect, with more accurate recall with the normal keypad display than with the random keypad or central presentation. In addition, recall of sequences with complex paths containing at least three crossings was consistently lower than recall of sequences with simple paths containing zero crossings. Observing a clear effect of path complexity confirms that presentation of the digit lists within a matrix-style display affords a visuo-spatial representation. However, path complexity affected recall in both the normal and random keypad conditions, and complex paths did not appear to remove the VSB effect observed with normal keypad presentation. While we could not decisively rule out an interaction between path complexity and presentation condition, if present, this interaction would reflect at most a moderation of the VSB effect when paths connecting the digits contain multiple crossings. Even with complex paths, the usual normal keypad advantage was apparent. Thus, both path complexity and presentation display influenced performance, possibly independently of each other. These results suggest that the VSB effect occurs even when visuospatial encoding is compromised.

Serial position analyses shed further light on these patterns. The VSB effect was concentrated upon the final items in the list. Indeed, our modelling suggested that recall of early-list items benefitted from presentation in distinct spatial locations even with the random layout. Performance in both the random and normal layouts exceeded that of central presentation at first, but for late items performance in the normal keypad condition exceeded performance in both other conditions. In contrast, we observed no evidence of any interaction between path complexity and serial position, but our evidence was insufficiently strong to discount this interaction.

The presence of clear path complexity effects in Experiment 1 confirms that visuo-spatial coding, in addition to verbal coding, operates in this task. However, verbal strategies, particularly chunking of digits, may shift reliance from visuospatial to verbal processes and hence lead participants to ignore path configurations, thereby obscuring potential effects of path complexity. If participants engaged frequently in chunking, then both the path complexity and visual-spatial boot-strapping effects we observed may have been diluted. Of the 34 participants in Experiment 1, 26 disclosed some form of strategy use. Fifteen explicitly reported verbalized chunking of digits, which could mean that any effects of path complexity, visuospatial bootstrapping, or an effect of path complexity on visuospatial bootstrapping may have been severely underestimated.

In Experiment 2, we disrupted the potential for verbal strategy use by requiring participants to engage in articulatory suppression (AS). AS is the repeated vocalization of a word or short phrase during a period of concurrent processing of verbal information. It is believed to disrupt both the formation and maintenance of verbal representations by occupying some of the same speech-motor architecture presumably needed for verbal rehearsal. Broadbent and Broadbent (1981) suggested that AS disrupts maintenance because without rehearsal, phonological representations are vulnerable to decay.

AS would be expected to disrupt any verbally-encoded information, including verbal encodings of visual images or spatial paths. AS may also impair memory for spatial information when spatial locations are intentionally bound to verbal information (Guérard, Tremblay, & Saint-Aubin, 2009; Morey, 2009). However, in the VSB paradigm, binding between digits and spatial location is incidental. In adding AS to our design, we presumed that participants’ ability to verbally rehearse would be limited and they would be compelled to rely more on the spatial processes thought to underpin the VSB effect. Previous evidence suggests that limiting verbalization with AS increases the VSB effect (Allen et al., 2015). Adding AS to our design should therefore reduce reliance on verbal strategy use, which might amplify any dependence on visual-path memory on producing the VSB effect, and therefore provide a better opportunity for observing differential costs of path complexity within familiar and random layouts.

## Experiment 2

### Method

***Participants.*** Twenty-nine students (5 male) were recruited in the same manner described in Experiment 1. Their ages ranged from 19 to 36 (*M* = 24.07, *SD* = 4.65). All had normal or corrected to normal sight, and were naive to the concept of visuospatial bootstrapping. Participants received an honorarium of £6 for partaking in the 50-minute session.

***Apparatus, stimuli and procedure.*** Experiment 2 was the same as Experiment 1, with the following exceptions. Participants were instructed to engage in concurrent AS during presentation of the stimuli. Because we expected that engaging in AS would diminish recall accuracy, we reduced the sequences from 7 to 6 digits. For sequences producing simple pathways, this involved removing the last digit from the sequence. For sequences producing complex pathways, it was essential to preserve as many crossings as possible. Often this could be effected by removal of the first or last digit from the sequence, but there were instances which demanded minor adjustments to the sequence’s route. The modified sequences are given in Appendix B. The average number of intersections remained similar in the normal (*M* = 3.08) and random (*M* = 3.16) keypad sequence sets.

Apart from these changes in stimuli, the procedure varied only slightly from that of Experiment 1. Participants were instructed to repeat aloud the word “Sunday” at a rate of approximately twice per second, commencing when the fixation cross appeared at the beginning of each trial and continuing until the recall prompt appeared. The researcher monitored compliance with these instructions.

### Results

***List-wise recall accuracy.*** We entered list-wise recall accuracies into a Bayesian ANOVA including display condition (central presentation, normal keypad, random keypad) and path complexity (simple, complex) as fixed factors, and participant identity as a random factor. The best model included effects of display condition and path complexity (*BF* = 682902, ±0.46%). Inclusion of the display condition (*BF* > 600) and path complexity effects (*BF* > 21) were strongly favored. Exclusion of their interaction was favored by a factor of 3.5.

Condition means are provided in Table 4. Numerically, the VSB effect was stronger with articulatory suppression in Experiment 2 than without it in Experiment 1. Compared with central presentation, the normal keypad display boosted recall accuracy by 13–21%. The numerical means offer no reason to suppose that the effect of path complexity differed in the normal and random keypad conditions; accuracy was 8% higher in the normal keypad condition with simple than complex paths and in the random keypad condition, 7% higher with simple paths.

**Table 4:** List-wise recall accuracy, Experiment 2.

Central Presentation	0.30 (0.25)	

	**Simple Path**	**Complex Path**

Normal Keypad	0.51 (0.28)	0.43 (0.28)
Random Keypad	0.40 (0.29)	0.33 (0.28)

*Note: N* = 29. Standard deviations in parentheses.

***Mean items per list correct.*** We entered mean number of items correct per list into a Bayesian ANOVA including the same factors. The best model included main effects of display condition and path complexity (*BF* = 2.38 × 10^20^, ±1.44%). Inclusion of the display condition (BF > 130) and path complexity effects (*BF* > 11) were convincing, as was exclusion of their interaction (*BF* > 12). Condition means may be found in Table 5.

**Table 5:** Mean number of correctly-recalled items per list, Experiment 2.

Central Presentation	4.02 (0.88)	

	**Simple Path**	**Complex Path**

Normal Keypad	4.90 (0.79)	4.60 (0.79)
Random Keypad	4.55 (1.00)	4.36 (1.02)

*Note: N* = 29. Standard deviations in parentheses.

**Figure 2:**
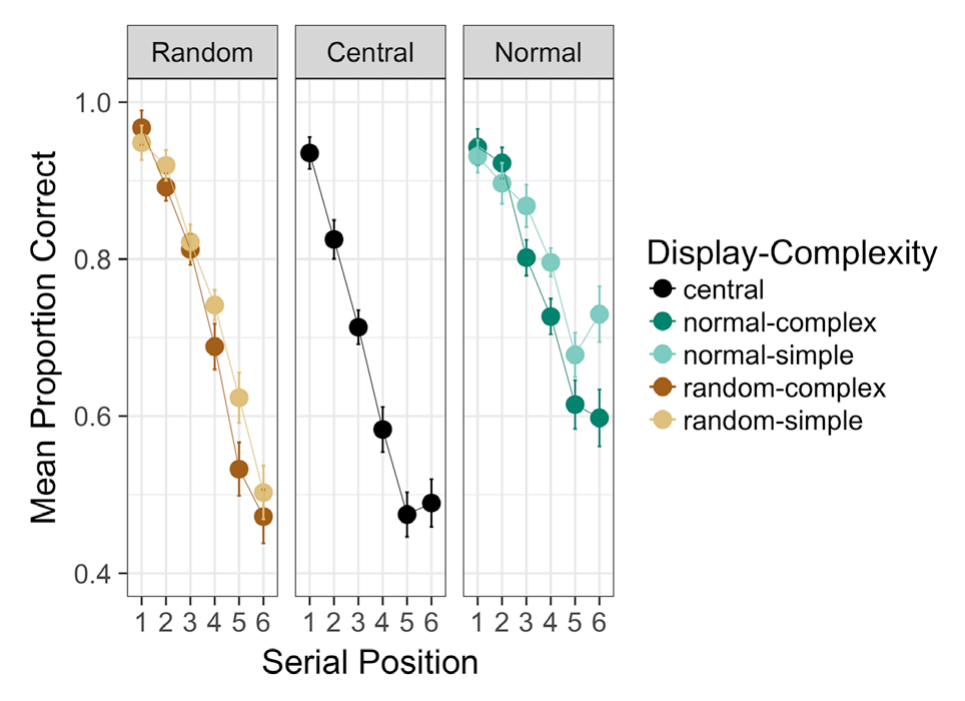
Experiment 2 serial position curves. Error bars are standard errors of the mean with the within-participants Cousineau-Morey correction applied. *N* = 29.

***Serial order analysis.*** Serial position curves are depicted in Figure 2. We entered proportions correct into a Bayesian ANOVA including fixed factors of display condition, path complexity, and serial position (1–6), along with a random effect of participant identity. The best model included each main effect plus interactions between serial position and display condition and serial position and path complexity (*BF* = 3.97 × 10^142^, ±1.02%). Inclusion of the interaction between path complexity and serial position was barely favored (*BF* = 1.30), but evidence decisively favored including the interaction between display condition and serial position (*BF* > 28,000). Inclusion of an effect of path complexity was decisively favored (*BF* > 200). Exclusion of an interaction between path complexity and display condition was favored by a factor of about 5.

We performed the same hypothesis tests as described in Experiment 1 to characterize the interaction between display condition and serial position. Condition means are provided in Table 6. As before, the best model distinguished between each level of display condition and included an interaction between display condition and simplified serial position, where positions 1–3 were considered “early-list” and positions 4–6 “late-list” (*BF* = 3.29 × 10^104^, ±1.38%). Inclusion of the interaction was favored by more than 60. The best model was favored over the next-best model with a simpler coding for display condition (in which central presentation differed from the others) by a factor of more than 3000. With a larger VSB effect, the need to distinguish between all presentation layouts was much clearer than it was in Experiment 1. All three were distinguished, and it is clear from the means in Table 6 that both the normal and random keypad displays were superior to central presentation early in the list. However, recall with the normal keypad display exceeded recall with the random display at the end of the list, though accuracy with the random display remained higher than with central presentation.

**Table 6:** Mean accuracy by display condition and list position, Experiment 2.

	Early-list	Late-list

Central Presentation	0.82 (0.16)	0.52 (0.23)

Normal Keypad	0.89 (0.12)	0.69 (0.24)
Random Keypad	0.89 (0.14)	0.59 (0.28)

*Note: N* = 29. Standard deviations in parentheses. We considered serial positions 1–3 early and positions 4–6 late.

### Discussion

In Experiment 2, we investigated the possibility that participants’ self-reported verbal strategy use in Experiment 1 may have diluted the effects of path complexity on the observed VSB effects by requiring participants in Experiment 2 to perform AS from start of encoding of to-be-remembered sequences until the point of recall. VSB effects were indeed larger in Experiment 2 under AS, consistently with Allen et al.’s (2015) results. Even with this substantially larger VSB effect, path complexity did not exert a larger effect on recall in the normal keypad than in the random keypad condition. Analysis of recall by serial position confirmed the results of Experiment 1 and their interpretation that the VSB effect and path complexity effects likely have different sources. Again, the VSB effect appeared for end-of-list items, while presentation in unique spatial locations boosted early list items even if the item-location mappings were unknown. We consider potential explanations for this clear, robust pattern in the General Discussion.

## General Discussion

The results from our two experiments corroborate current evidence for the visuospatial bootstrapping phenomenon and add novel information helpful for explaining why it occurs. In addition to manipulating the manner in which the digits were displayed, we manipulated the complexity of the path that digit presentation generated through the random and normal keypad displays, testing whether complex paths attenuated the VSB effect. We compared recall with normal keypads with both central and random keypad presentations. Across two experiments we replicated the VSB effect, in which verbal recall was superior with the normal keypad display compared with the random keypad display or central presentation. We always found effects of path complexity on memory for digits, regardless of the familiarity of the keypad display. Complex paths resulted in reductions in accuracy of 7–8% with both keypad displays. These findings show that spatial path information, which should be incidental to performance of a verbal recall task, was generated during visual presentation and impaired verbal recall. However, the familiarity of the spatial display did not determine whether a visual-spatial path was generated nor whether it affected verbal recall, because path complexity impaired recall in the random keypad condition as much as in the normal keypad condition. The VSB effect therefore does not occur *because* the familiar display uniquely affords the production of a spatial memory trace, nor because the familiar display uniquely encourages participants to adopt a visuospatial encoding strategy to better their performance. Indeed, we observed clear recall benefits in the random keypad condition compared to central presentation under AS, suggesting that though knowledge of consistent verbal-spatial mappings is useful, verbal serial recall may also benefit from spatially representing unfamiliar layouts.

Serial position effects on the other hand systematically differed by display condition. In both experiments, it was clear that the VSB effect really emerged for late-list items. Though performance in the normal keypad condition was superior to performance with central presentation throughout the list, performance in the random keypad condition also enjoyed an early-list advantage relative to central presentation. Recall in the normal keypad condition clearly exceeded recall in the random condition only for late-list items. This pattern cannot be attributed to our participants learning the static random keypad display we used. Participants were asked to reproduce the positions of the digits in the static random keypad at the end of the session, but only one participant (from Experiment 1) out of the 63 we tested did so accurately. There is therefore no reason to suspect that the same knowledge-based “bootstrapping” phenomenon known to occur with normal keypad displays partially occurred in the random keypad condition. Instead, these serial position curves suggest that two distinct factors underlie the VSB effect that occurs with normal keypad presentation. Presentation within a matrix layout affords the production of a distinct visuospatial representation, and our path complexity effects confirm that this occurs regardless of knowledge of any consistent mapping between the verbal content and spatial locations. However, this visuospatial representation may be of limited use without prior knowledge of the mappings between spatial and verbal content. Possibly, the boost we observed primarily for early-list items in the random versus the central display condition represents encoding of a few novel digit-location bindings. In the normal keypad condition, perhaps early-list improvement is also attributable to encoding of digit-location bindings, but explicit knowledge of the digits’ locations in the keypad allows participants to deduce digit identity from the end of the spatial path representation, propelling recall benefits through the end of the list.

We have replicated and confirmed Allen et al.’s (2015) finding that the VSB effect increases under the influence of concurrent AS, but in contrast, our visuospatial manipulation did not clearly attenuate the VSB effect. Inclusion of complex path crossings impaired recall in the random keypad condition as much as in the normal keypad condition. Allen et al. did not include a random keypad control condition in their design, and doing so might have changed their interpretation somewhat. Based on our path complexity manipulation and our interpretation of the robust interactions between display condition and serial position, we would expect imposing concurrent spatial tapping during encoding to impair recall in the random as well as the normal keypad condition. Such a pattern might be observed if we assume that spatial tapping prevents the formation of a visuospatial representation. However, it is even more likely that spatial tapping does not prevent the formation of a spatial trace, but rather prevents the use of it. Parmentier and Andrés (2006) found that while concurrent spatial tapping reduced spatial memory performance overall, it did not change the size of the effect of path crossings. This suggests that complexity and spatial tapping exert different effects on spatial memory, and that spatial tapping does not prevent spatial memories from forming. Applied to the VSB paradigm, we think path complexity alters the quality of the spatial memory, but does not prevent the spatial representation from influencing verbal recall, whereas spatial tapping (and perhaps various other concurrent tasks) prevents participants from utilizing the spatial memory representation to influence their spoken response. The essence of the “bootstrapping” advantage is not the generation of the spatial representation alongside any verbal representation, but rather the advantageous use of multi-modal encoding.

Our finding of a clear path complexity effect on verbal serial recall with random keypad displays contrasts with the findings of Guérard et al. (2013), who found no evidence that path complexity impaired verbal serial recall or single-item probed recall. A likely explanation for this discrepancy lies in the difference between the consistency and predictability of the spatial displays between the two investigations. In our studies, the same restricted set of 10 locations presented within a structured matrix were always used. In contrast, Guérard et al.’s participants encountered randomly-selected locations without any planned structure. Because structured paths are known to be more memorable than unstructured ones (Kemps, 2001), perhaps the paths generated in our study were memorable enough for participants to utilize when planning their recall sequence. There are also salient differences in the mode of recall between Guérard et al.’s paradigm and ours that may affect whether incidental spatial memories are used in the service of verbal recall. To equate responding in their verbal and spatial recall conditions, Guérard et al. (2013) required participants give their verbal responses via mouse. The letter choices were displayed linearly at the foot of an otherwise blank screen for the participant to choose in correct serial order. Finding the response choices within this display may have resulted in the participant generating a new spatial path representation, potentially overwriting any representation formed earlier during encoding of the spatially-located letters. With spoken recall in our investigation, there would have been no overt visual stimulus to afford interference with the previously-generated path.

How might the contribution of visuospatial representations to visuospatial bootstrapping as revealed by the effects of path complexity be explained by different models of working memory? Traditionally, explanations of the VSB effect have invoked the multi-component working memory model (Baddeley, 2000; 2012). Current versions of the multi-component model include distinct storage modules specifically for verbal and visuospatial information, a domain-general episodic buffer capable of holding information retrieved from long-term memory or from either temporary storage buffer, and a central executive responsible for allocating attention. Applying the model to VSB phenomena, during these verbal recall tasks a phonological representation of the visually-presented digit list is generated and maintained in the phonological loop, a representation of the spatial path is maintained in the visuospatial sketchpad, and representations containing both features are held in the episodic buffer. Dual-task investigations have supported some of these contentions. Concurrent articulatory suppression, believed to selectively impair the phonological loop, impairs verbal recall overall, but not the VSB effect specifically (Allen, et al., 2015). Imposition of spatial tapping, believed mainly to affect the functioning of the visuospatial sketchpad by disrupting the maintenance of a visual path representation, annihilates the VSB effect if performed during encoding (Allen, et al., 2015). The presumption that an episodic buffer is important for the VSB phenomena has not been confirmed by explicit manipulation. Rather, it is assumed because it is clear that long-term knowledge of the digit-location mappings affects the emergence of VSB effects.

Our novel results can be explained in term of the multi-component working memory model, but relying on the multi-component model to comprehensively explain all the visuospatial bootstrapping phenomena results in a somewhat awkward explanation. Our finding of path complexity effects in both matrix-style display conditions confirms that a representation of the visuospatial path is generated when presentation affords it, and we could surmise that this representation is retained separately from any verbal representation that is also rendered. The limited benefit of mere dual-encoding of verbal and visuospatial traces, and the necessity for linking them together and furthermore connecting relevant long-term knowledge with them could indeed be explained by invoking an episodic buffer capable of combining verbal and visuospatial representations and of integrating long-term knowledge with novel memoranda. However, we cannot argue that the multi-component framework is uniquely capable of explaining our VSB findings, nor that it necessarily offers the best explanation. It is noteworthy that the multi-component model’s progenitors acknowledge that neither the phonological loop nor the visuospatial sketchpad incorporate mechanisms adequate for the construction, retention, and retrieval of representations of serial order (Hurlstone, et al., 2014), which is what is measured in the VSB paradigm. This naturally poses problems for invoking the multi-component model for explaining the occurrence of the VSB effect. Furthermore, the idea that verbal and visuospatial memories are maintained in both domain-specific and integrated domain-general forms within the multi-component framework hinders, rather than facilitates, a straightforward account of the novel VSB phenomena we discovered. According to the latest account of how information accesses the episodic buffer, relevant contents of the verbal and visual modules are fed into the episodic buffer for integration with each other or with retrieved long-term knowledge (Baddeley, Allen, & Hitch, 2011). Applied to visuospatial bootstrapping, verbal and spatial traces are recorded in their specialized modules and in the episodic buffer, where they may be integrated. When verbal-spatial mappings are familiar, this integration is perhaps then facilitated by long-term knowledge, also made available by the episodic buffer. Notwithstanding evidence inconsistent with Baddeley et al.’s (2011) proposition that visuospatial information is simultaneously represented in domain-specific and domain-general forms (Morey, Guérard, & Tremblay, 2013), this account places immense explanatory burden on an episodic buffer. The episodic buffer is assumed to flexibly integrate information from at least three sources, and moreover, because it does not necessarily do this whenever information of all types is available (e.g., Guérard et al., 2013), we must also suppose some sort of selectivity. Thus, though we *can* describe how our findings may be situated with a multi-component working memory framework, it is awkward both to assume a mechanism as flexible as the episodic buffer and to suppose that verbal and visuospatial representations are necessarily held simultaneously in two distinct forms.

For a model of working memory to explain these VSB phenomena, it is not necessary to propose that verbal and visuospatial memories are maintained separately, nor that both domain-specific and integrated representations are generated. It is necessary to allow for dual-encoding of multi-modal aspects of the stimuli. However, a variety of research findings already demands this of a working memory model (e.g., Logie, Della Sala, Wynn, & Baddeley, 2000; Logie, Saito, Morita, Varma, & Norris, 2016), and we can think of no up-to-date working memory model that does not allow for domain-specificity in some manner. Such representational flexibility is assumed in embedded process models of working memory, where the contents of the focus of attention and activated long-term memory may take any form (e.g., Cowan, 2005; Oberauer, 2002, 2009). The same flexibility would also arise via domain-specific perceptual and motor mechanisms according to gestural-perceptual accounts of short-term memory phenomena (e.g., Macken, Taylor, & Jones, 2015). The VSB phenomena demonstrate that short-term memory performance is facilitated by relevant long-term knowledge (e.g., Ericsson & Kintsch, 1995) and multi-modal representations are generated by visual presentation of verbal materials, but the VSB phenomena do not necessarily require the multi-component framework, and do not obviously falsify other models of working memory.

In conclusion, our results confirm that visuospatial representations and long-term knowledge may both boost serial recall in an ostensibly verbal task. Our results limit the way in which we presume visuospatial representations assist verbal recall. It is not the case that VSB effects occur because familiar spatially arranged layouts uniquely afford the creation of helpful spatial memories. Instead, spatial representations apparently emerge when the stimulus presentation affords them, and exert some benefits on verbal serial recall regardless of whether correspondence between the verbal items and spatial layout is known. In the context of the VSB paradigm, these benefits appear to be focused on early-list items. Knowledge of consistent verbal-spatial mappings particularly boosts end-of-list recall, allowing further benefits to emerge.

## Data Accessibility Statement

Anonymized data and materials associated with this project may be found on the Open Science Framework (https://osf.io/9g5sv/). DOI: https://doi.org/10.17605/OSF.IO/9G5SV

## Additional Files

The Additional files for this article can be found as follows:

10.5334/joc.2.s1 Appendix A. Digit Sequences, Experiment 1.

10.5334/joc.2.s2 Appendix B. Reduced Digit Sequences, Experiment 2.

## Author Information

A.A. generated the idea for this research, and C.C.M. and S.D. joined A.A. in designing the first experiment. All co-authors contributed to deciding to add articulatory suppression in the second experiment. A.A. conducted both experiments, recorded and coded the data. We are grateful to Mou Yiqing and Meagan Freeman for assistance with data collection and coding. A.A. and C.C.M. collaborated on data analysis and writing the earliest draft, which the other co-authors helped to improve. C.C.M. prepared the data analysis supplement, which is available at https://osf.io/9g5sv/ with the data and other materials. A.A. and C.C.M. are grateful to Women in Working Memory (WomWoM) for encouragement. Address correspondence to Candice C. Morey, School of Psychology, 70 Park Place, Cardiff, CF10 3 AT, United Kingdom. Email: MoreyC@cardiff.ac.uk.
