# Supplementary material for: On the Right Track? Investigating the Effect of Path Characteristics on Visuospatial Bootstrapping in Verbal Serial Recall
Source: J Cogn. 2017 Dec 8;1(1):3. doi: 10.5334/joc.2 (PMC6644921; doi:10.5334/joc.2)
Supplement: Appendix A. — Digit Sequences, Experiment 1. [file joc-1-1-2-s1.docx]

Appendix A: Digit Sequences, Experiment 1


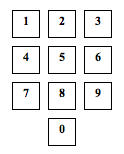
Normal keypad

Complex Simple

6148237 2473806

7316925 0459632

3792516 1548709

3719405 0975612

0381654 4870612

5679102 9057431

5927046 6874253

2615473 7048369

3721054 3164987

5876429 5290817

0163527 3654908

5941068 6027413


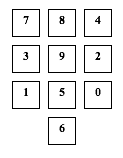
Random keypad

Complex Simple

0738952 2098356

8601234 9520617

1482357 8394205

6734891 5984206

8610372 3195028

0352198 1924873

2375146 4295387

5417283 8795604

4085732 2073156

0821694 7842051

1947680 6598731

1084236 0948735
