# Supplementary material for: On the Right Track? Investigating the Effect of Path Characteristics on Visuospatial Bootstrapping in Verbal Serial Recall
Source: J Cogn. 2017 Dec 8;1(1):3. doi: 10.5334/joc.2 (PMC6644921; doi:10.5334/joc.2)
Supplement: Appendix B. — Reduced Digit Sequences, Experiment 2. [file joc-1-1-2-s2.docx]

Appendix B: Reduced Digit Sequences, Experiment 2


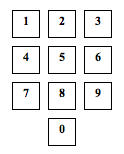
Normal Keypad

Complex Simple

148237 247380

731692 045963

379256 154870

371940 097561

038164 487061

679102 905743

927046 687425

261543 704836

372104 316498

876429 529081

163257 365490

594106 602741

Random Keypad


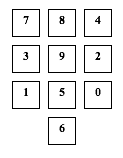
 Complex Simple

073852 209835

860123 952061

148235 839420

673489 598420

861034 391502

035219 192487

237514 420538

541728 879560

085732 207315

082694 784205

147680 659873

108326 094873
